# Towards treatment planning and treatment of deep-seated solid tumors by electrochemotherapy

**DOI:** 10.1186/1475-925X-9-10

**Published:** 2010-02-23

**Authors:** Damijan Miklavcic, Marko Snoj, Anze Zupanic, Bor Kos, Maja Cemazar, Mateja Kropivnik, Matej Bracko, Tjasa Pecnik, Eldar Gadzijev, Gregor Sersa

**Affiliations:** 1 University of Ljubljana, Faculty of Electrical Engineering, Trzaska cesta 25, SI-1000 Ljubljana, Slovenia; 2 Institute of Oncology Ljubljana, Zaloska 2, SI-1000 Ljubljana, Slovenia

## Abstract

**Background:**

Electrochemotherapy treats tumors by combining specific chemotherapeutic drugs with an intracellular target and electric pulses, which increases drug uptake into the tumor cells. Electrochemotherapy has been successfully used for treatment of easily accessible superficial tumor nodules. In this paper, we present the first case of deep-seated tumor electrochemotherapy based on numerical treatment planning.

**Methods:**

The aim of our study was to treat a melanoma metastasis in the thigh of a patient. Treatment planning for electrode positioning and electrical pulse parameters was performed for two different electrode configurations: one with four and another with five long needle electrodes. During the procedure, the four electrode treatment plan was adopted and the patient was treated accordingly by electrochemotherapy with bleomycin. The response to treatment was clinically and radiographically evaluated. Due to a partial response of the treated tumor, the metastasis was surgically removed after 2 months and pathological analysis was performed.

**Results:**

A partial response of the tumor to electrochemotherapy was obtained. Histologically, the metastasis showed partial necrosis due to electrochemotherapy, estimated to represent 40-50% of the tumor. Based on the data obtained, we re-evaluated the electrical treatment parameters in order to correlate the treatment plan with the clinical response. Electrode positions in the numerical model were updated according to the actual positions during treatment. We compared the maximum value of the measured electric current with the current predicted by the model and good agreement was obtained. Finally, tumor coverage with an electric field above the reversible threshold was recalculated and determined to be approximately 94%. Therefore, according to the calculations, a small volume of tumor cells remained viable after electrochemotherapy, and these were sufficient for tumor regrowth.

**Conclusions:**

In this, the first reported clinical case, deep-seated melanoma metastasis in the thigh of the patient was treated by electrochemotherapy, according to a treatment plan obtained by numerical modeling and optimization. Although only a partial response was obtained, the presented work demonstrates that treatment of deep-seated tumor nodules by electrochemotherapy is feasible and sets the ground for numerical treatment planning-based electrochemotherapy.

**Trial registration:**

EudraCT:2008-008290-54

## Background

Electrochemotherapy is a type of tumor treatment that combines the use of specific chemotherapeutic drugs which have an intracellular target and low membrane permeability, with application of electric pulses to the tumors to increase drug uptake into cells. It provides good local tumor control when the two modalities combined are optimized; *i.e*. drug choice, distribution and concentration in the tumors, in addition to adequate electric pulse parameter selection and pulse delivery leading to cell membrane electroporation of the tumor tissue [1-4].

The drug used in electrochemotherapy needs to be adequately distributed in the tumor and present at a sufficient concentration. For treatment of small subcutaneous tumor nodules, such as melanoma metastases, intratumoral bleomycin or cisplatin administration is recommended, whereas for treatment of bigger tumor nodules, intravenous injection of bleomycin is used. Drug doses needed for treatment are provided in the published Standard Operating Procedures [5]. For optimal drug distribution within the tumor after intratumoral injection, only a few minutes are needed between the drug injection and electroporation of tumors. After intravenous bleomycin injection, at least 8 minutes are needed for the drug to be in a pharmacological peak in the tumor and the drug remains at a sufficient concentration for at least another 20 minutes [6].

The second prerequisite for successful electrochemotherapy is that the whole tumor mass is exposed to a sufficiently high electric field. This can be achieved by appropriate selection and placement of electrodes, and application of electric pulses of adequate amplitude. The distribution of the electric field after application of electric pulses by plate or needle electrodes has already been extensively elaborated for small tumor nodules [2]. These settings and electrodes provide efficient treatment of superficial tumor nodules up to 3 cm in diameter in a single electrochemotherapy session and have even been used to treat bone cancer [7-9]. However, to enable treatment of deep-seated tumors, a design of long needle electrodes and in particular their positioning with respect to the tumor is needed. If solid tumors of 3-4 cm diameter are located deep in the body, choosing electrical parameters that would result in a good clinical response and that would have no or minimal damage to normal tissue is of the outmost importance, especially in cases where tumors are located close to vital organs. Numerical modeling in treatment planning is the proposed approach that also allows verification of the electrical parameters based on the clinical response to electrochemotherapy [10].

In order to also develop electrochemotherapy for treatment of deep-seated tumors, the aim of our study was to treat a deep-seated melanoma metastasis in the thigh of a patient by custom-made long needle electrodes. Based on treatment planning, the electric pulse parameters and positioning of electrodes were determined. The patient was treated accordingly by electrochemotherapy with bleomycin. The response to treatment was clinically, radiographically and histologically evaluated. Due to a partial response of the treated tumor (reduction in size by more than 50%), the metastasis was surgically removed after 2 months and pathological analysis was performed. Based on the data obtained, we re-evaluated the electrical treatment parameters in order to correlate the treatment plan with the clinical response of the electrochemotherapy-treated metastasis.

## Materials and methods

### Clinical data of the patient

**Figure 1:**
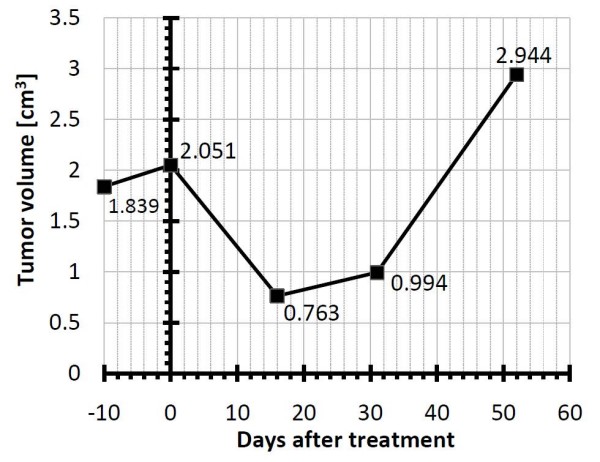
**Size of the melanoma metastasis determined by ultrasound**. The figure shows the tumor size shortly before the treatment, at the time of the treatment itself and during the follow-up. Regrowth of the tumor was observed at day 31 and the tumor was excised at day 52.

A 51-year-old male Caucasian patient with a diagnosis of melanoma had been previously treated by electrochemotherapy with bleomycin given intravenously. The treated small superficial metastases on the right leg regressed completely after the treatment. In October 2008, a PET-CT was performed, which revealed a deep-seated metastasis in the right thigh (Figure 1). In December 2008, electrochemotherapy with long needle electrodes was performed using bleomycin given intravenously (15,000 IU/m^2^) and electric pulses were delivered 10-12 minutes after injection. The study was approved by the national Ethics committee and institutional board. The patient signed an informed consent to participate in the study. The positioning of the electrodes was ultrasonographically guided. The treatment was performed in general anesthesia; in order to avoid strong muscle contractions induced by electric pulses the myorelaxant vercuronium bromide (Norcuron, Organon) was used. In February 2009, regrowth of the metastasis was observed by ultrasound. The metastasis was surgically removed by the end of February and a pathological analysis of the excised tissue was performed.

### Numerical treatment planning

Based on the PET-CT radiographs, the position of the melanoma metastasis was determined. Its size was 20 × 14 mm in diameter and it was located in the right thigh, 20 mm under the skin. The location was estimated to be on or minimally invasive into thigh muscle fascia.

**Figure 2:**
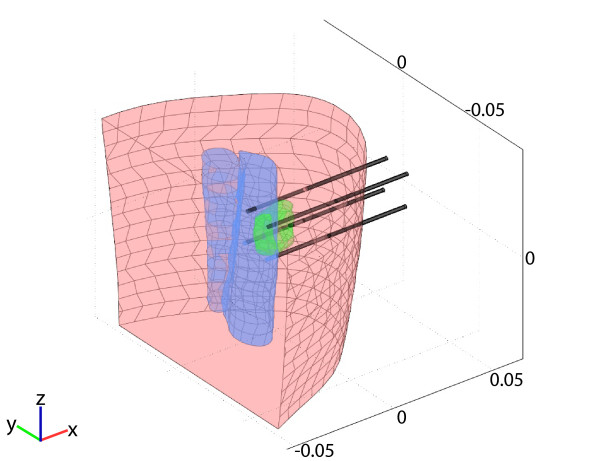
**Anatomical model consisting of adipose tissue (pink), muscle tissue (blue), melanoma metastasis (green) and inserted electrodes**. Note that only the closest two muscles were considered in the model, as the other muscle groups lying further away would not contribute to the model accuracy and would only increase the computational cost.

During preparation for electrochemotherapy, a slight growth of the metastasis was observed ultrasonographically (Figure 1). The anatomical model geometry used in the treatment planning procedure was obtained from CT images. All clinically relevant tissue structures (tumor, muscle, adipose tissue) were delineated and used to construct 3D geometry objects in the numerical computing environment Matlab (Mathworks, USA) by the planar contour method as previously described by [11] (Figure 2). The geometry of objects was then imported into the finite element analysis software Comsol Multiphysics 3.5 (COMSOL AB, Sweden). In the model, all tissues were considered homogeneous, the assigned conductivity values being: 0.2 S/m for the tumor, 0.018 S/m for adipose tissue, 0.135 S/m and 0.75 S/m for muscle tissue perpendicular and parallel to the muscle direction, respectively. These conductivity values were considered as approximations for DC values, and are extrapolated from measurements performed at 10 Hz. During the delivery of electric pulses, the conductivity of tissues changes as a consequence of electroporation. Measurements of conductivity during pulse application have shown that conductivity changes by a factor of around 3.5; therefore conductivity of electroporated tissue was increased by this factor, which agrees well with measurements taken on rat muscle and liver tissue and with the post-electroporation conductivity estimation performed by mathematical modeling for rabbit liver [12,13]. All conductivity values were chosen according to previous measurements of tumor and tissue conductivity and models of subcutaneous tumor and skin electroporation [12-14].

Numerical calculations were performed with Comsol. Electric field distribution in the tissue, caused by an electroporative pulse, was determined by solving the Laplace equation for static electric currents:

where σ and φ are tissue electric conductivity and electric potential, respectively. The boundary conditions used in our calculations were a constant potential on the surface of the electrodes and electrical insulation on all outer boundaries of the model. For tumors, the reversible electroporation threshold was considered to be 400 V/cm, for adipose tissue 100 V/cm, for muscle tissue 200 V/cm and 80 V/cm, in the perpendicular direction to muscle fibers and in the parallel direction, respectively. The irreversible threshold was set to 900 V/cm for all tissues.

In treatment planning, a numerical model of electroporation was used that did not take into account changes in tissue conductivity during electroporation. After the treatment, in order to compare the measured electric currents during electric pulse delivery and the currents predicted by the model, a model that took into account the changes in tissue conductivity was used. The conductivity dependencies on the electric field σ(E) of all tissues were approximated by a step function [14].

Electrode positions and voltages to be applied between individual electrodes were optimized using a genetic algorithm, described in more detail in previous studies [15,16]. In short, the algorithm optimized the position of each electrode (x, y, z) - in discrete steps of 1 mm and the voltage between each pair of electrodes in discrete steps of 100 V. Feasible ranges of all these parameters were taken into account, as well as the specifications of the electric pulse generator (see below). First, a population of treatment plans (consisting of all electrode positions and all used voltages) was randomly generated. The treatment plans then evolved over several hundred generations by mathematical operations cross-over and mutation according to the fitness function:

where F is fitness, V_Trev _is the tumor volume subjected to the local electric field above the reversible threshold and V_Hirrev _is the volume of healthy tissue subjected to a local electric field above the irreversible threshold. The weights in the fitness function were chosen to reflect the importance of the individual parameters for efficient ECT. Namely, V_Trev _is crucial for efficient ECT; therefore its weight is larger (100) than the weight of V_Hirrev_. The algorithm was stopped after 500 iterations - this number of iterations gave good solutions in previous studies [15,16] and the quality of the treatment plan was compared to previously set treatment plan requirements - when a sufficiently high quality treatment plan was achieved, *i.e*. the whole tumor was covered with a sufficiently high electric field and very little of the surrounding tissue was affected by the field. The optimization took 5 h on a Windows XP, 3.0 GHz, 2 GB RAM desktop computer.

### Electrodes, pulse generator and pulse parameters

Four custom-made electrodes made of stainless steel, 1.8 mm in diameter with sharpened tips, insulated except for the upper 4 cm, were used. An additional 1.2 mm in diameter electrode was considered for direct insertion into the center of the tumor. The electrodes were connected to independently controlled generator outputs of the Cliniporator Vitae (IGEA, Carpi, Italy). The Cliniporator Vitae device is a pulse generator with 6 independently controlled and electrically insulated outputs each providing up to 3000 V, max current 50 A, delivering 8 rectangular electrical pulses (rise time 1 μs) of 100 μs duration at a pulse repetition frequency of 4 Hz [17]. The current and voltage are measured and logged with a precision better than 3%, which allows for pulse delivery control and post-treatment evaluation. Pulses were delivered 10-12 minutes after i.v. bolus injection of bleomycin.

## Results

### Optimized treatment plan

**Figure 3:**
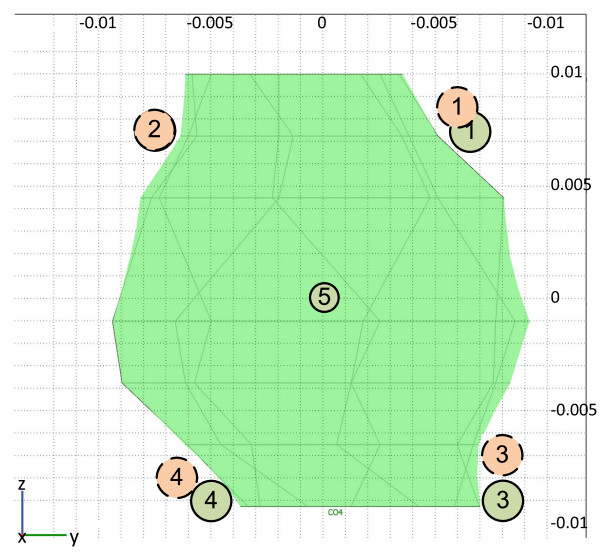
**Electrode positions according to the treatment plan**. Electrode positions for four (dashed line circles) and five (solid line circles) electrodes are shown. The presented tumor cross-section is a parallel projection of the tumor on a plane parallel to the skin surface. Note that electrode 2 is in the same location in both treatment plans.

Treatment planning was performed for two different electrode configurations. The first configuration was with five electrodes with a central electrode inserted in the tumor and four electrodes distributed around the tumor, while the second configuration was with four electrodes outside the tumor (Figure 3). Although the five-electrode option was recognized as superior and was the primary choice for treatment, the central electrode could not be inserted into the tumor as the tumor was very mobile, effectively "floating" in the surrounding adipose tissue. This mobility of the tumor also made it very difficult to rigorously follow the treatment plan for four electrodes, and as a result the electrodes were positioned farther away from the tumor than originally planned.

The results of optimization were electrode positions and minimum voltages for each electrode pair. Electrode positions outside the tumor were similar in both configurations, *i.e*. very close to the tumor surface; however the fifth electrode inside the tumor significantly reduced the required voltage to achieve efficient membrane electroporation of cells in the whole tumor, thereby also reducing damage to healthy tissue. The maximum voltages required were 3000 V and 2500 V in the four and five electrode configuration, respectively. The volume of irreversibly permeabilized healthy tissue according to the treatment plan was 13.8 cm^3 ^(of that 11.5 cm^3 ^adipose tissue and 2.33 cm^3 ^muscle tissue) in the four electrode configuration and 12.3 cm^3 ^(of that 10.4 cm^3 ^adipose tissue and 1.88 cm^3 ^muscle tissue) in the five electrode configuration. Depth of insertion was a few millimeters deeper than the tumor, slightly penetrating the muscle tissue.

### Treatment and response to the treatment

**Figure 4:**
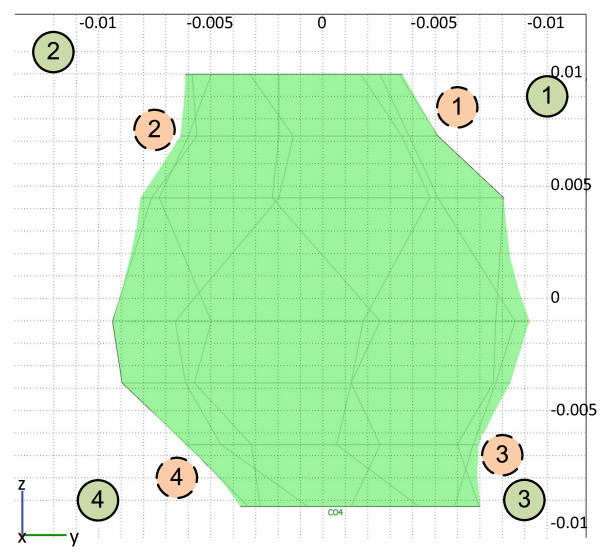
**Actual positioning of the electrodes relative to the treatment plan**. Electrode positions according to the treatment plan (dashed line circles) and actual electrode positions during treatment as determined by ultrasonography and photo documentation (solid line circles). The presented tumor cross-section is a parallel projection of the tumor on a plane parallel to the skin surface.

**Figure 5:**
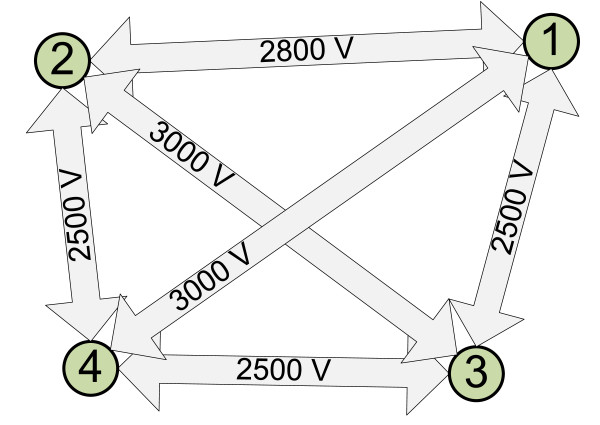
**Combinations of electric pulse delivery between the electrodes as well as voltages between each pair**.

During the procedure, the four-electrode treatment plan was adopted. The electrodes were placed according to the treatment plan as depicted in Figure 4. Electrodes were positioned under ultrasonographic guidance in the four outer corners of the tumor in the fat tissue, the deepest location being minimally inserted into the muscle (Figure 4). Eight pulses of 100 μs each were delivered between each pair of electrodes. In total, 6 times 8 pulses were delivered to the tumor with amplitudes of 2800 V between electrodes (pair 1-2) 25 mm apart, 2500 V between electrodes (1-3, 2-4 and 3-4) 20 mm apart, and 3000 V between diagonal electrodes (1-4 and 2-3) (Figure 5). The currents recorded during electric pulse delivery were between 9 and 19 A.

The treatment was performed in general anesthesia and due to the myorelaxant given to the patient, only minor muscle contractions were observed. No other side-effects were noticed. The patient reported no discomfort after the treatment, and left the hospital after 2 days. The response to treatment with electrochemotherapy was followed ultrasonographically at regular time intervals (Figure 1). The first post-operative ultrasound showed a substantial decrease in the tumor volume (more than 50%), while the second showed a regrowth of tumor tissue.

**Figure 6:**
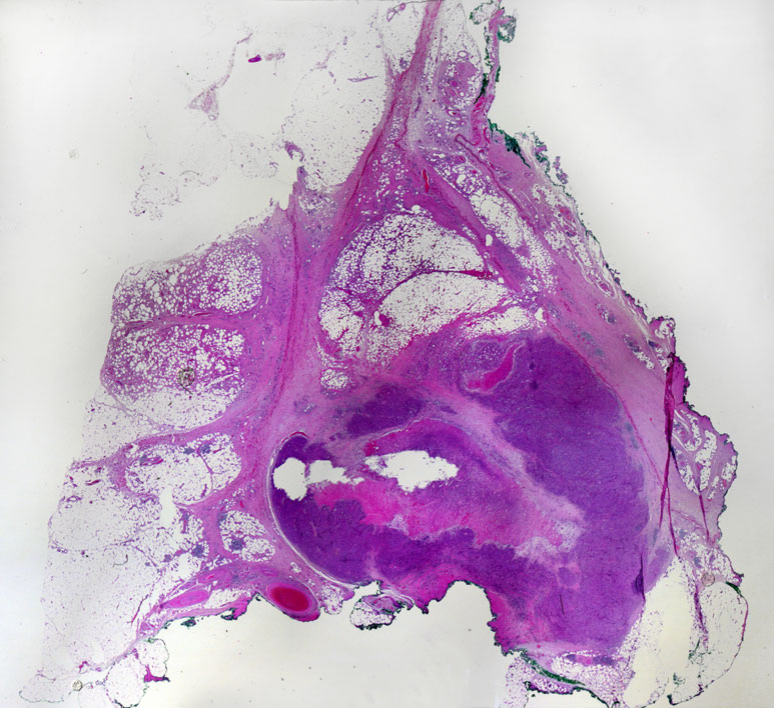
**Cross-section of the excised melanoma metastasis (*in toto*)**.

In February 2009, *i.e*. 52 days after electrochemotherapy was performed, the metastasis was excised. It was located 2 cm under the skin in the deep subcutaneous fat tissue, abutting on the muscle fascia. The size of the metastasis measured after excision (22 × 15 mm) was determined on the pathological cross-section (Figure 6).

**Figure 7:**
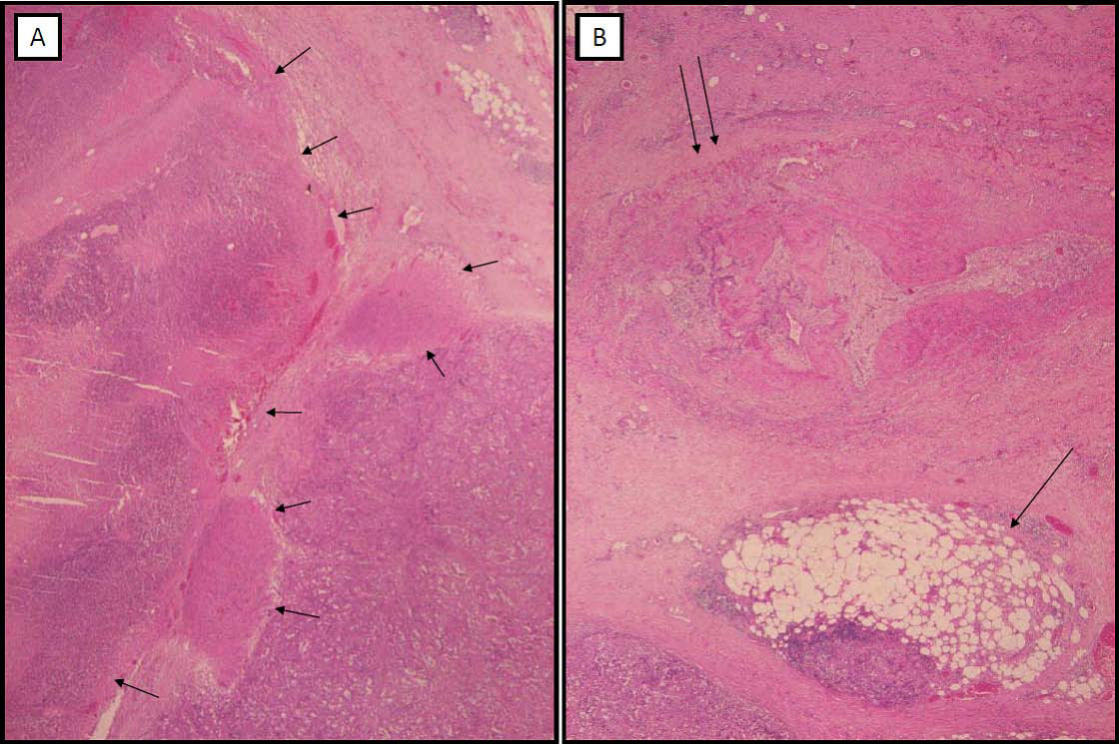
**Histology of melanoma metastasis treated by electrochemotherapy**. The tumor (A) shows partial necrosis (short arrows). In the surrounding tissue (B), fat necrosis (long arrow) and obliterated blood vessels (double arrow) are visible (H&E, original magnification 20×).

Histologically, the metastasis showed partial necrosis, estimated to represent 40-50% of the tumor. It was not possible to discriminate between spontaneous and induced necrosis. However, there was indirect evidence of the effect of electrochemotherapy; *i.e*. the presence of fat necrosis and obliterated blood vessels in the tissue around the tumor (Figure 7). These observations would not be expected in a fast-growing, untreated metastasis.

### Numerical model validation

**Figure 8:**
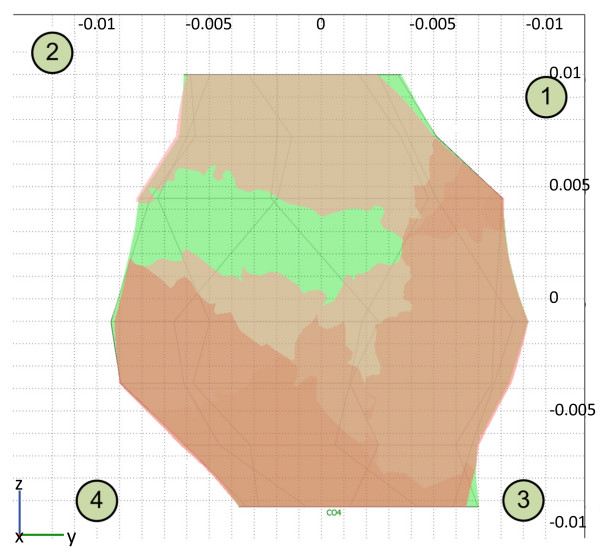
**Prediction of tumor permeabilization**. The figure shows the numerical prediction of tumor permeabilization after application of all electric pulses in a plane parallel to the skin surface, passing through the centre of the tumor. The green color represents unpermeabilized tumor tissue, while shades of brown represent tissue permeabilized by one or more pulse sequences (from a lighter to a darker shade). The cross-section is made through the centre of mass of the tumor. At this location, the tumor has the largest cross-section area and therefore coverage is less than 94%.

After the treatment, the geometry of the numerical model was updated according to measurements taken during the operating procedure and photo documentation of the treatment. Specifically, the four electrode positions in the model were changed according to these measurements (Figure 4). We compared the maximum value of the electric current measured by the Cliniporator Vitae during electric pulse delivery with the current predicted by the numerical model. Good agreement was obtained between the measurements and calculations, as presented in Table 1. Finally, tumor coverage with an electric field above the reversible threshold was recalculated using the revised geometry and the volume of the reversibly permeabilized tumor was determined to be approximately 94% (Figure 8).

**Table 1:** Agreement between maximum measured electrical currents and currents calculated in the numerical model

Electrode pair	Measured current [A]	Calculated current [A]	Error [%]
1-2	16	16.2	1
1-3	18	17.3	-4
1-4	10	9.5	-5
2-3	19	17.2	-9
2-4	9	8.4	-7
3-4	9	8.0	-11

## Discussion

We present here the first study of deep-seated tumor electrochemotherapy based on numerical treatment planning. Namely, electrochemotherapy was, until now only used for superficial and accessible tumor nodules, with an approximately 80% objective response rate [1,7,18-22]. In this, the first reported clinical case, a deep-seated melanoma metastasis in the thigh of the patient was treated by insertion of long needle electrodes around the tumor. A new electric pulse generator was used that provides higher voltages and currents and has six independently controlled insulated outputs, which thus allow for treatment of deeper-seated tumors by delivering electric pulses between different pairs of electrodes.

The electric conductivity values taken into account in the numerical model have been obtained from measurements done mostly on large animal tissues, which are not necessarily valid for human tissue as well [13]. Furthermore, these values are not valid for electroporated tissue, as electroporation increases tissue conductivity. In one of our previous studies, we measured the conductivity of rat muscle and liver tissue and the conductivity increased between 3.2-3.8 times for both tissue types [12]. Also, comparing the measured and calculated electric current for rabbit liver resulted in an estimated conductivity increase of 3.6 [11]. In this study, an increase in conductivity of 3.5 as a result of electroporation was used for all tissues, a choice which seems to be at least partly validated by the agreement between the measured and calculated electric current in our study (Table 1).

In our post-treatment model, tissue electroporation thresholds for muscle and tumor were taken from previous studies in which these thresholds were determined by comparing *in vivo *measurements and numerical modeling of electroporation of different tissues [14,23,24]. In all these studies, the assumption was made that the values of the electric field, at which a change in tissue properties occur, coincides with the electroporation thresholds. This assumption was already considered both theoretically and practically in previous studies and can be considered as justified [25-27].

Two different electroporation models were used in our present study; the first which took into account changes in tissue properties and the second which did not, a simplification that made the calculation much faster. Both models predicted similar electroporation volumes (results not shown), while only the first model could predict the electric current density. As such, the second model was used for optimization-based treatment planning and the first one for validation by comparing the measured and calculated electric currents.

According to the model used after the treatment, inaccuracies in positioning of the electrodes are most likely responsible for the inadequate electroporation of the entire tumor volume, although possible deviation from the assigned electrical conductivities and/or deviations from the assigned electroporation threshold for the tissues cannot be disregarded. Nevertheless, good agreement between the predicted and the measured delivered currents implies that the conductivity values chosen were very close to the real values. Under the assumption that the positioning of the electrodes was responsible for the inadequate tumor electroporation, calculations show that only a small percent of the tumor was not successfully electroporated (app. 6%); however this was enough for the tumor to survive and start growing again. While normally these relatively small errors in electrode positioning (Figure 4) would not lead to an unsuccessful treatment, the proximity of three different tissues with very different conductivities made the treatment very sensitive to electrode positioning. Adipose tissue that surrounded the tumor had by far the lowest conductivity, which meant that according to the voltage divider principle the electric field was largest in the adipose tissue around the tumor and not in the tumor [28].

## Conclusions

Electrochemotherapy of a deep-seated tumor was performed in a patient with the aim to verify the treatment approach, and the use of treatment planning in optimizing the positioning of the electrodes and electrical parameters. Although the configuration of five electrodes was recognized as the best in treatment planning, it was not possible to execute it due to "floating" of the tumor in the adipose tissue. The four-electrode position was thus used and at follow-up of the tumor growth, significant tumor reduction was observed (Figure 1). For effective treatment, however, all viable tumor cells have to be destroyed in order to prevent tumor regrowth. Therefore, as calculated in this tumor model, even a small percentage of remaining viable tumor tissue (6%) after electrochemotherapy was enough for tumor regrowth. Nevertheless, this clinical case demonstrates that treatment of deep-seated tumor nodules by electrochemotherapy is feasible and that optimization of the treatment approach by tumor numerical modeling is of significant help.

## Competing interests

The authors declare that they have no competing interests.

## Authors' contributions

DM, MS, MC, MK, TP, EG and GS were involved in treatment of the patient. MB performed pathology of the metastasis. DM, AZ and BK carried out medical image segmentation and performed numerical calculations. DM, AZ, BK and GS prepared the manuscript. All authors read and approved the final manuscript.
